# Gender Differences in the Inheritance Mode of RYR2 Mutations in Catecholaminergic Polymorphic Ventricular Tachycardia Patients

**DOI:** 10.1371/journal.pone.0131517

**Published:** 2015-06-26

**Authors:** Seiko Ohno, Kanae Hasegawa, Minoru Horie

**Affiliations:** 1 Cardiovascular and Respiratory Department, Shiga University of Medical Science, Otsu, Japan; 2 Center for Epidemiologic Research in Asia, Shiga University of Medical Science, Otsu, Japan; 3 Department of Cardiovascular Medicine, Kyoto University Graduate School of Medicine, Kyoto, Japan; 4 Department of Cardiovascular Medicine, University of Fukui Faculty of Medical Science, Eiheiji, Japan; Indiana University, UNITED STATES

## Abstract

Catecholaminergic polymorphic ventricular tachycardia (CPVT) is one of the causes of sudden cardiac death in young people and results from RYR2 mutations in ~60% of CPVT patients. The inheritance of the RYR2 mutations follows an autosomal dominant trait, however, *de novo* mutations are often identified during familial analysis. In 36 symptomatic CPVT probands with RYR2 mutations, we genotyped their parents and confirmed the origin of the respective mutation. In 26 sets of proband and both parents (trio), we identified 17 *de novo *mutations (65.4%), seven from their mothers and only two mutations were inherited from their fathers. Among nine sets of proband and mother, five mutations were inherited from mothers. Four other mutations were of unknown origin. The inheritance of RYR2 mutations was significantly more frequent from mothers (n = 12, 34.3%) than fathers (n = 2, 5.7%) (*P* = 0.013). The mean ages of onset were not significantly different in probands between *de novo* mutations and those from mothers. Thus, half of the RYR2 mutations in our cohort were *de novo*, and most of the remaining mutations were inherited from mothers. These data would be useful for family analysis and risk stratification of the disease.

## Introduction

Catecholaminergic polymorphic ventricular tachycardia (CPVT) is an inherited disease characterised by polymorphic ventricular tachycardia induced by exercise or emotional stress in childhood[1]. Sometimes, the first attack of CPVT leads to sudden cardiac death in the young. Five causative genes of CPVT have been reported; RYR2[2], CASQ2[3], KCNJ2[4], TRDN[5] and CALM1[6], and >60% of CPVT patients carry mutations in RYR2[7]. RYR2 gene encodes cardiac ryanodine receptor (RyR2), and RyR2 is indispensable for Ca^2+^ release from sarcoplasmic reticulum (SR), consequently controls the cardiac contraction[8]. CPVT-related RYR2 mutations were reported to cause abnormal calcium leak from SR[9].

The inheritance mode of CPVT is both autosomal dominant and recessive; mutations in RYR2[2], KCNJ2[4] and CALM1[6] follow a dominant trait, CASQ2[3] and TRDN[5], recessive. Among RYR2-positive CPVT patients, large CPVT families with RYR2 mutations have been reported in the literature, however, sporadic cases have also been frequently found. We recently demonstrated two families with RYR2 exon 3 deletion[10]. In both families, the mutation was inherited from the maternal side, and there was only one male among six carriers of this exon deletion mutation. As the inheritance mode or frequency of *de novo* RYR2 mutation has not been extensively studied, this study searched for the characteristics of RYR2 mutations in the view of their mode of inheritance.

## Methods

### Study Cohort

**Fig 1:**
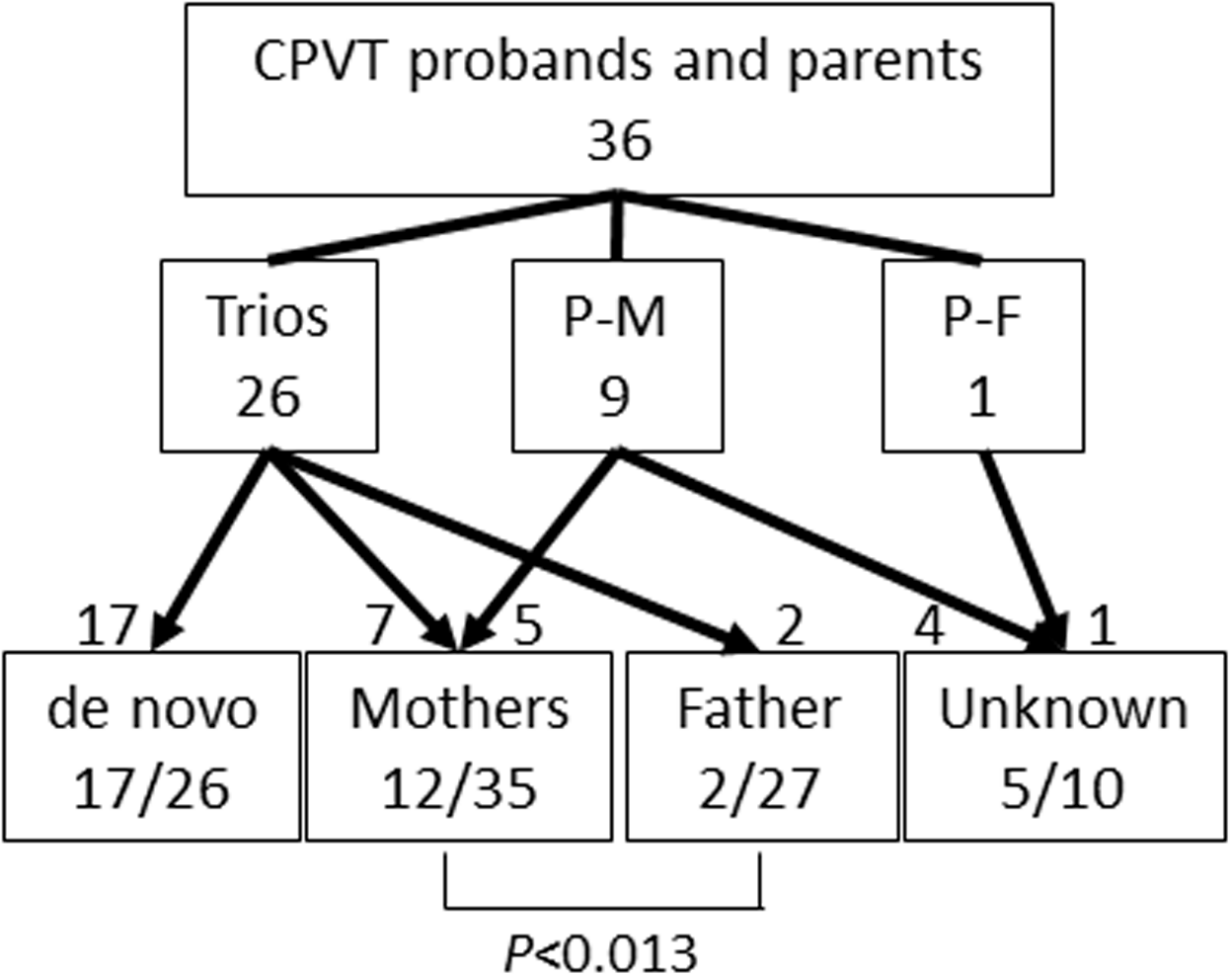
Scheme for Mutation Inheritance. Showing the number of screened family members and the origin of RYR2 mutations. The boxes in the middle lane show genotyped family members in each group. Trio; proband and both parents, P-M; proband and mother, P-F; Proband and father.

The study cohort consisted of 36 Japanese CPVT probands (18 boys) with RYR2 mutations, their 62 parents and their 29 siblings. All the probands suffered syncope or cardiac pulmonary arrest (CPA) and were registered for genetic screening between 2005 and 2013 in Shiga University of Medical Science. Genetic analysis was performed after obtaining written informed consent in accordance with the study protocol approved by the Institutional Review Board of Shiga University of Medical Science. The approved number is 23–128. In the study protocol, we included the statement that the research results would be published with anonymized clinical information. If the participants were minors or children, we obtained the informed consent verbally from them and written consent from their guardians. The verbal consent was recorded in the clinical record, and our Institutional Review Board approved to obtain written consent from their guardians. According to the participating family members, we divided them into three groups: 26 sets of proband and both parents (trio), 9 sets of proband and mother (P-M) and 1 sets of proband and father (P-F) (Fig 1). All the participating parents were also genotyped, and the origin of the mutation was classified into four groups: *de novo*, from mother, from father and unknown. Their consanguinity of *de novo* group were confirmed by screening of 15 single nucleotide polymorphisms and 1 microsatellite. The unknown group included the case where the inheritance mode could not be determined due to the non-participation of either one of their parents. We evaluated the clinical and genetic characteristics of the probands and compared them in two groups: *de novo* and maternal origin. The mutation locations were classified into three groups based on the previous report [11]. In 23 trio families, we compared the age of parents at the birth of the probands in three groups: *de novo*, maternally- and paternally originated mutations.

### Statistical analysis

All continuous variables are reported as mean ± SD. Differences between continuous variables were evaluated using unpaired Student *t*-test or Mann-Whitney Rank Sum Test, and categorical variables were analysed using Fisher exact test. Statistical significance was considered at *P*<0.05.

## Results

### Origin of the mutations

**Fig 2:**
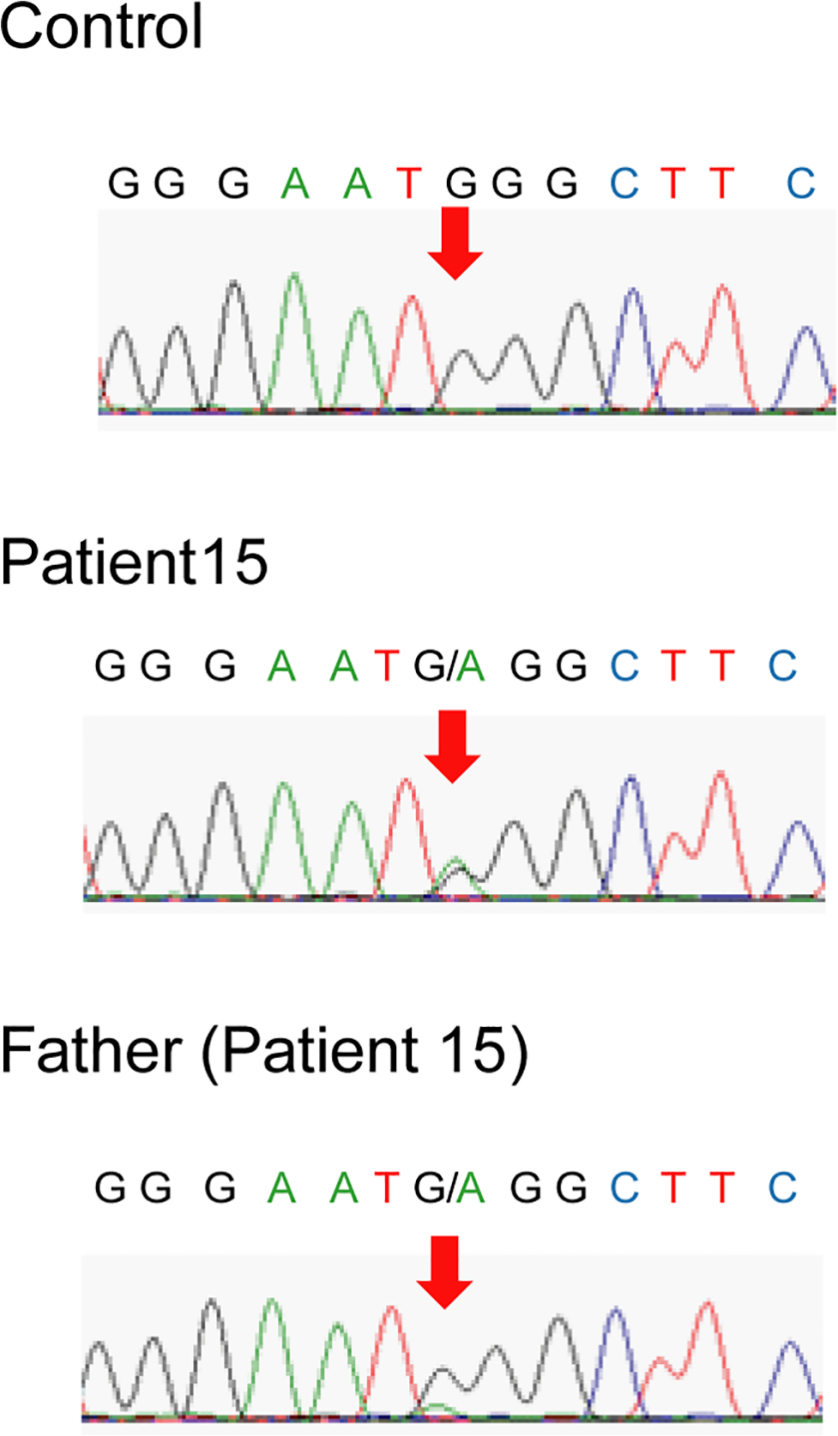
Sequence Electropherograms of Patient 15. Sequence electropherogram of control (upper), patient 15 (middle) and his father (bottom). The peak of the mutant ‘a’ allele in 7024 residue (red arrow) was lower than the original ‘g’ peak in the sequence electropherogram of the father, which suggested the presence of mosaicism.

In 26 mutations identified in 26 probands of the trio group, 17 RYR2 mutations were confirmed to be *de novo*, seven mutations were from their mothers and two from their fathers. (Fig 1 and Table 1). One of the fathers (patient 15) was suspected to carry the mutation in mosaicism (Fig 2). Five mutations from nine mutations in the P-M group were inherited from their mothers, and four were unknown origin; *de novo* or from their fathers. Although four fathers in the P-M group did not agree to the genetic analysis, they were all healthy and had no history of syncope or cardiac arrest. The mother and maternal grandmother of patient 26 (Table 1) died suddenly at a young age, and his sister carried the same mutation. Accordingly, the mutation appeared to come from his mother side, but we failed to obtain their maternal genomic information. Therefore, his mutation was consequently classified as unknown origin.

**Table 1:** Clinical and genetic summaries of probands.

		Age			RYR2 mutation					Phenotype of Parents	
Patient Number	Sex	Genetic Analysis	Onset	Most severe symptom	Nucleotide	Amino Acids	Location	Genotyped Family Members	Inheritance	Father	Mother
1	F	17	16	Syncope	exon 3 deletion	N57_G91del35	NT	Trio	Maternal	none	AF
2	F	11	9	Syncope	exon 3 deletion	N57_G91del35	NT	P-M	Maternal	none	syncope
3	F	9	9	Syncope	506g>t	R169L	NT	Trio	de novo	none	none
4	F	5	5	CPA	506g>a	R169Q	NT	Trio	de novo	none	none
5	F	9	9	CPA	506g>a	R169Q	NT	Trio	de novo	none	none
6	M	16	16	CPA	533g>c	G178A	NT	Trio	de novo	none	none
7	M	13	11	Syncope	1221a>t	R407S	NT	P-M	de novo or F	none	none
8	F	12	7	CPA	1259g>a	R420Q	NT	P-M	Maternal	none	syncope
9	M	3	3	Syncope	3667a>g	T1223A	NT	Trio	Maternal	none	none
10	F	11	11	Syncope	3766c>a	P1256T	NT	Trio	Maternal	none	none
11	F	15	12	Syncope	4552c>t	L1518F	NT	Trio	Maternal	none	none
12	F	25	10	Syncope	5170g>a	E1724K	NT	P-M	Maternal	none	syncope
13	M	13	13	CPA	6574a>t	M2192L	Central	Trio	Maternal	none	none
14	M	13	13	CPA	6737c>t	S2246L	Central	Trio	de novo	none	none
15	M	14	14	Syncope	7024g>a	G2342R	Central	Trio	Paternal (mosaic)	none	none
16	M	11	10	CPA	7169c>t	T2390I	Central	Trio	Paternal	none	none
17	M	15	10	CPA	7199g>t	G2400V	Central	Trio	de novo	none	none
18	M	12	12	CPA	7423g>t	V2475F	Central	P-M	de novo or P	none	none
19	F	18	8	CPA	11583g>c	Q3861H	Central	Trio	de novo	none	none
20	F	8	8	Syncope	11583g>t	Q3861H	Central	Trio	de novo	none	none
21	F	27	6	Syncope	11836g>a	G3946S	Central	P-M	de novo or P	none	none
22	F	16	6	Syncope	11836g>a	G3946S	Central	Trio	de novo	none	none
23	F	28	28	CPA	11917g>a	D3973N	Central	Trio	Maternal	none	none
24	M	3	3	Syncope	12006g>a	M4002I	Central	Trio	de novo	none	none
25	M	9	9	Syncope	12371 g>a	S4124N	CT	P-M	Maternal	none	none
26	M	11	11	CPA	12458g>t	S4153I	CT	P-F	de novo or M	none	SD
27	M	11	2	Syncope	12533a>g	N4178S	CT	Trio	de novo	none	none
28	F	6	6	CPA	12579c>g	C4193W	CT	Trio	de novo	none	none
29	M	10	10	Syncope	13463a>c	Q4488P	CT	Trio	de novo	none	none
30	F	33	9	Syncope	13798t>c	F4600L	CT	Trio	de novo	none	none
31	M	28	10	Syncope	14174a>g	Y4725C	CT	Trio	de novo	none	none
32	F	23	9	Syncope	14311g>a	V4771I	CT	P-M	Maternal	none	syncope
33	M	13	13	CPA	14311g>a	V4771I	CT	P-M	de novo or P	none	none
34	M	17	14	CPA	14806c>a	Q4936K	CT	Trio	de novo	none	none
35	M	5	5	CPA	14834_14835insTCA	4944_4945insH	CT	Trio	de novo	none	none
36	M	12	5	CPA	9910c>g, 14222c>t	Q3304E, A4741V	Central and CT	Trio	Maternal	none	syncope

CPA; cardiac pulmonary arrest, NT; N-terminal, CT; C-terminal, SD; sudden death

In total, 17 mutations were confirmed to be *de novo* from the 26 trio group, 12 were from 35 mothers and only two were from 27 fathers (Fig 1). The frequency of mutations originating from mothers was significantly higher than that from fathers (*P* = 0.013). Five of 12 mothers with mutations suffered syncope and were diagnosed as CPVT after the diagnosis of their children. In contrast, no father had symptoms nor was diagnosed as CPVT (Table 1).

We extended the genetic analysis in the 29 siblings of the probands from 21 families; 14 in *de novo*, 9 in maternal origin, one in paternal origin and 5 in unknown mutation origin. We identified 4 siblings with mutations in maternal origin families and one sibling with a mutation in a paternal origin family, though there were no genotype positive siblings in families of probands with *de novo* mutation. The result suggested the low possibility of germline mosaicism in *de novo* families.

### Location of mutations

Among 12 mutations inherited from mothers, seven (58.3%) were located in the N-terminus, while only four (23.6%) from 17 *de novo* mutations were located in the N-terminus (Table 1). Regarding four *de novo* N-terminal mutations, three were at residue 169. In contrast, two maternal mutations (16.7%) were located in the central domain and three (12.5%) were located in the C-terminus. Both paternal mutations were located in the central domain.

### Clinical comparison between de novo and the P-M group

We compared clinical characteristics between 17 probands with *de novo* mutations and 12 with mutations from the maternal side (Table 2). In the *de novo* mutation group, nine probands (52.9%) were male, while in the maternal mutation group there were only four males (33.3%). The mean ages of onset were younger in *de novo* mutation carriers (8.4 ± 3.6 y.o.) than in mother oriented mutation carriers (11.0 ± 6.4 y.o.). There was no significant difference in the severity of symptoms between the two groups.

**Table 2:** Clinical characteristics of probands with *de novo* or maternal mutations.

	*de novo*	Maternal
	n = 17	n = 12
Male n (%)	9 (52.9)	3 (27.2)
Mean age of Onset	8.4±3.6	11.0±6.4
CPA n (%)	9 (52.9)	4 (33.3)
Syncope n (%)	8 (47.1)	7 (66.7)

### Ages of parents at birth of probands

**Fig 3:**
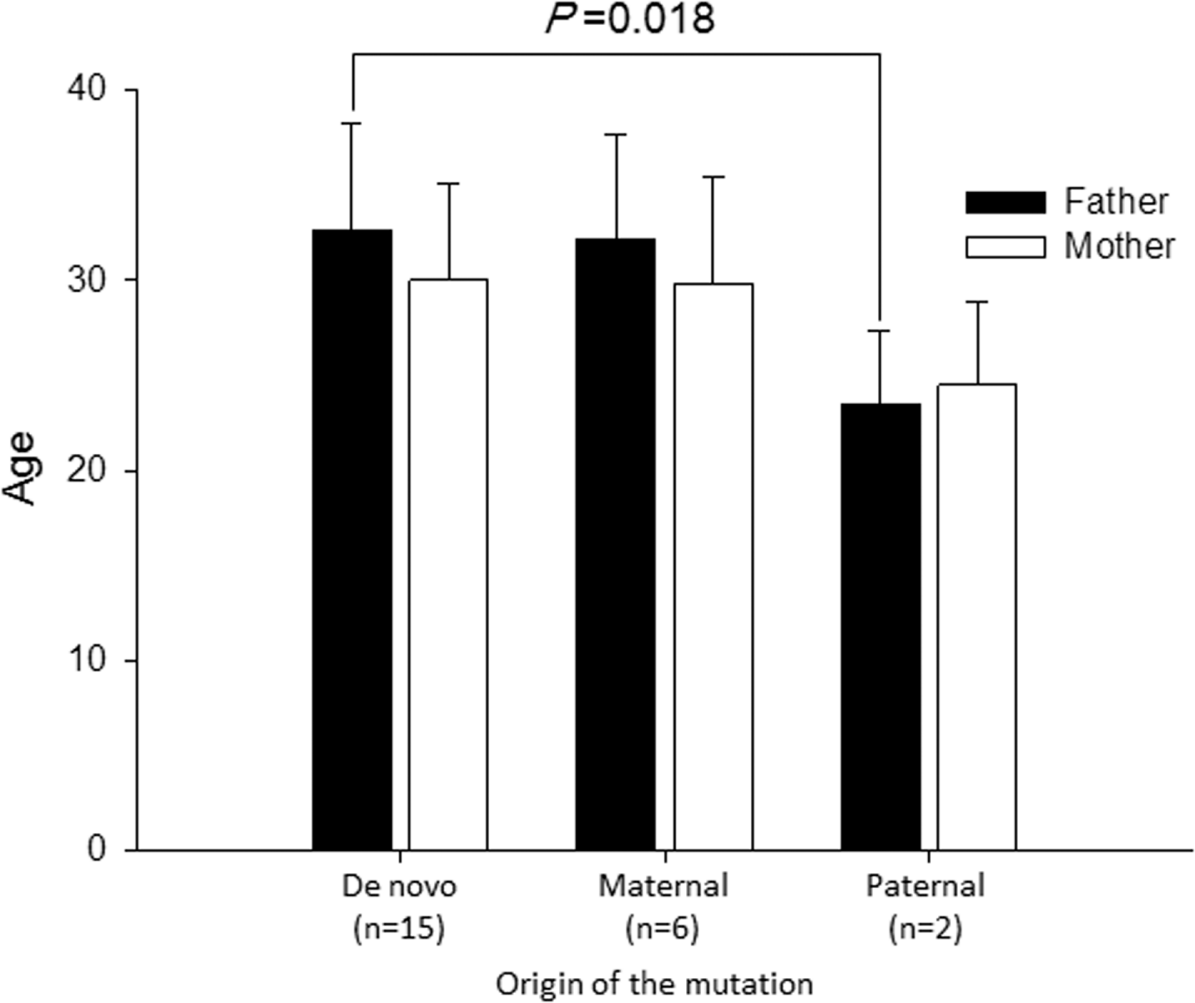
Mean Age of parents depends on the RYR2 mutation origin. Bar graphs depict mean ages of parents at the birth of probands. Filled bars indicate those of fathers and open bars those of mothers. The mean age of genotype-positive fathers was significantly younger than that of the *de novo* mutation group.

In 23 trio families, we examined the ages of parents at the births of probands (Fig 3). According to the category of RYR2 mutations, there were 15 *de novo*, six maternal and two paternal origin. The mean age of fathers carrying mutations was significantly younger than that in the *de novo* group (23.5 ± 4.9 y.o. vs. 32.6 ± 5.3 y.o. *P* = 0.019). On the other hand, there was no difference of the ages among mothers.

## Discussion

In the present study, we first demonstrated that almost half of CPVT-related RYR2 mutations were *de novo*, and the remaining were mostly inherited from mothers. Although the inheritance of CPVT caused by RYR2 mutations follows an autosomal dominant trait, the high frequency of *de novo* mutations would result in higher occurrence of sporadic cases and thereby confusing the precise diagnosis of CPVT.

One of the most well-known *de novo* mutations is FGFR3-G380R, which is detected in more than 98% of patients with achondroplasia [12]. Advanced paternal age has been shown to increase the risk for the disease, and germline mutation in sperm has been reported in the disease. In RYR2 mutations, we showed that the age of fathers in the *de novo* mutation group was older than that of fathers with mutations, though the number of paternal inheritance was small, and mosaicism may be present in one of the fathers.

Several reports have investigated the inheritance of RYR2 mutations identified in CPVT. Priori et al.,[13] demonstrated that RYR2 mutations were identified in 14 families, and 10 of these 14 mutations were *de novo* and two were inherited from mothers and only one was inherited from the father. The remaining one mutation was probably inherited from the maternal side because she died suddenly at 38 years old. Notably, two genotype-positive mothers in their report were symptomatic, while one genotype-positive father was not. Thus, there was a possibility that the paternal mutation was not the major cause of CPVT, just a rare variant. In our study, two genotype-positive fathers were also asymptomatic, and their mutation sites were very close (residue 2342 and 2390).

In contrast, a dissimilar inheritance pattern was reported in 2012[11]. In familial evaluation with RYR2 mutations, 17 families confirmed the inheritance of RYR2 mutations; six in both *de novo* and maternal, and five in paternal. In the report, we could not obtain the phenotype of their parents or confirm the malignancy of mutations; however, the high paternal mutation inheritance differed from that reported in the previous study.

The low frequency of paternal inheritance may result from the poor prognosis of male patients compared to females[13]. Male CPVT patients might die before the age of reproduction. Indeed, we found that fathers of probands with paternal RYR2 mutations were younger than those of probands with *de novo* mutations. Recently, flecainide therapy for prevention of polymorphic ventricular tachycardia has been prevalent[14, 15], and adequate ICD implantation will improve the prognosis of CPVT patients. These therapies may change the inheritance mode of RYR2 mutations in CPVT in the future.

### Study Limitation

Although we reported that half of the RYR2 mutations identified in CPVT were *de novo*, we could not completely rule out cases of mosaicism by PCR-based Sanger methods. In addition, we could not search for the germline mosaicism using other organs nor next generation sequencing analysis to detect low frequency mosaicism.

## Conclusions

Almost half of RYR2 mutations identified in CPVT patients were *de novo*, and others were mainly inherited from their mothers. Parents with RYR2 mutations often remain asymptomatic, therefore we need a strict and detailed history taking, exercise tolerance test and genetic survey to prevent a severe cardiac phenotype occurring in younger mutation carriers and their siblings.
