# Correction: Gender Differences in the Inheritance Mode of RYR2 Mutations in Catecholaminergic Polymorphic Ventricular Tachycardia Patients

**DOI:** 10.1371/journal.pone.0243476

**Published:** 2021-02-19

**Authors:** Seiko Ohno, Kanae Hasegawa, Minoru Horie

In the Abstract, there is an error in the sixth sentence. The correct sentence is: The inheritance of RYR2 mutations was significantly more frequent from mothers (n = 12, 34.3%) than fathers (n = 2, 7.4%) (P = 0.015). In the Origin of the mutations subsection of the Results, there is a similar error in the second sentence of the second paragraph. The correct sentence is: The frequency of mutations originating from mothers was significantly higher than that from fathers (P = 0.015).

There are several errors in the Location of mutations subsection of the Results. The correct paragraph is: Among 12 mutations inherited from mothers, seven (58.3%) were located in the N-terminus, while only four (23.5%) from 17 de novo mutations were located in the N-terminus (Table 1). Regarding four de novo N-terminal mutations, three were at residue 169. In contrast, two maternal mutations (16.7%) were located in the central domain and two (16.7%) were located in the C-terminus. One mother carried two mutations in the Central and C-terminus.

In the Ages of parents at birth of probands subsection of the Results, the P value of the age difference in fathers between de novo and paternal is incorrectly reported as 0.019. The correct P value is 0.037.

There are errors in Table 1 and Table 2. Please see the correct tables here.

There are errors in Fig 1 and Fig 3. Please see the correct figures here.

**Fig 1:**
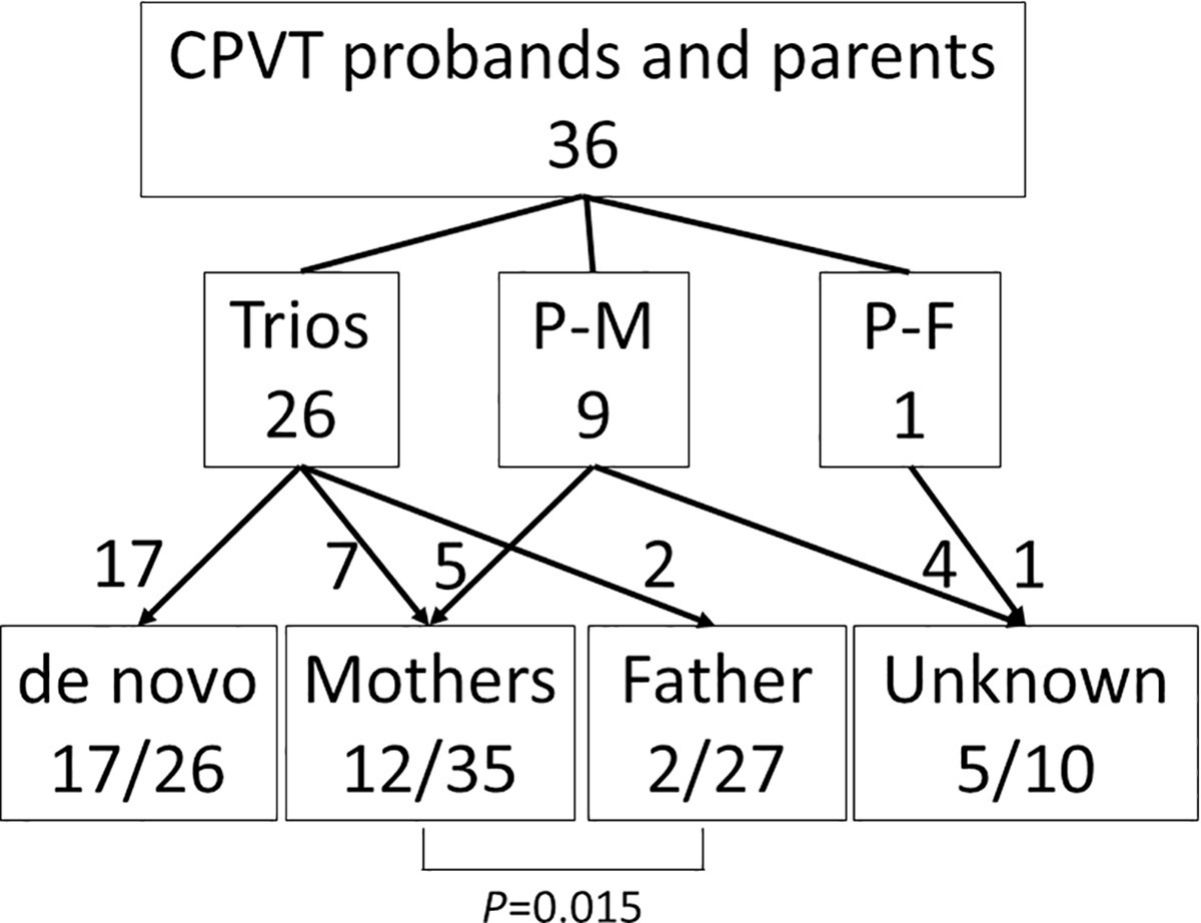
Scheme for Mutation Inheritance. Showing the number of screened family members and the origin of RYR2 mutations. The boxes in the middle lane show genotyped family members in each group. Trio; proband and both parents, P-M; proband and mother, P-F; Proband and father.

**Fig 3:**
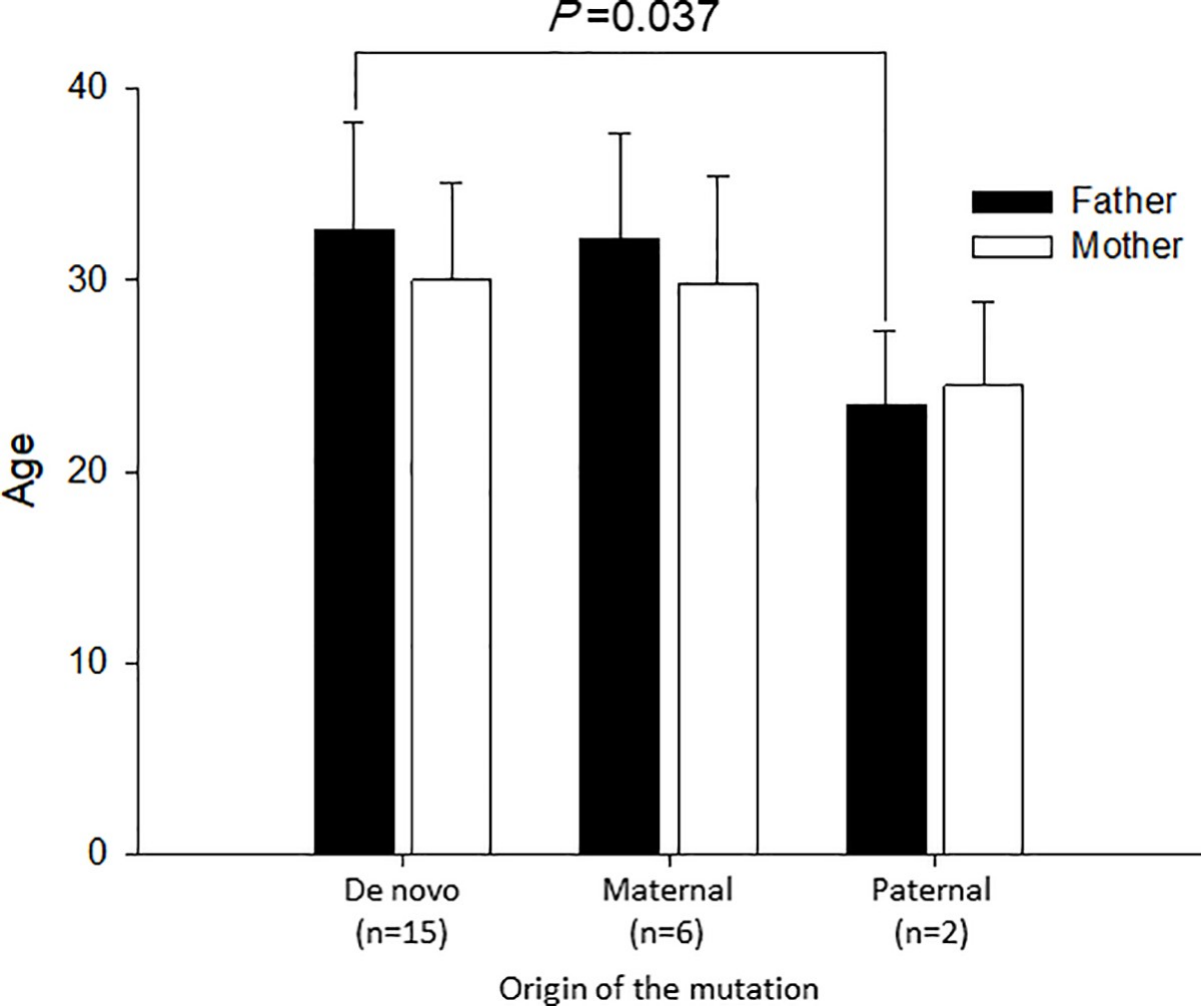
Mean Age of parents depends on the RYR2 mutation origin. Bar graphs depict mean ages of parents at the birth of probands. Filled bars indicate those of fathers and open bars those of mothers. The mean age of genotype-positive fathers was significantly younger than that of the de novo mutation group.

**Table 1:** Clinical and genetic summaries of probands.

Patient Number	Sex	Age		Most severe symptom	RYR2 mutation			Genotyped Family Members	Inheritance	Phenotype of parents	
		Genetic Analysis	Onset		Nucleotide	Amino Acids	Location				
										Father	Mother
1	F	17	16	syncope	exon 3 deletion	N57_G91del35	NT	Trio	Maternal	none	AF
2	F	11	9	syncope	exon 3 deletion	N57_G91del35	NT	P-M	Maternal	none	syncope
3	F	9	9	syncope	506g>t	R169L	NT	Trio	de novo	none	none
4	F	5	5	CPA	506g>a	R169Q	NT	Trio	de novo	none	none
5	F	9	8	CPA	506g>a	R169Q	NT	Trio	de novo	none	none
6	M	16	12	CPA	533g>c	G178A	NT	Trio	de novo	none	none
7	M	13	11	syncope	1221a>t	R407S	NT	P-M	de novo or F	none	none
8	F	12	7	CPA	1259g>a	R420Q	NT	P-M	Maternal	none	syncope
9	M	3	3	syncope	3667a>g	T1223A	NT	Trio	Maternal	none	none
10	F	11	5	syncope	3766c>a	P1256T	NT	Trio	Maternal	none	none
11	F	15	12	syncope	4552c>t	L1518F	NT	Trio	Maternal	none	none
12	F	25	10	syncope	5170g>a	E1724K	NT	P-M	Maternal	none	syncope
13	M	13	13	CPA	6574a>t	M2192L	Central	Trio	Maternal	none	none
14	M	13	13	CPA	6737c>t	S2246L	Central	Trio	de novo	none	none
15	M	14	11	syncope	7024g>a	G2342R	Central	Trio	Paternal (mosaic)	none	none
16	M	11	10	CPA	7169c>t	T2390I	Central	Trio	Paternal	none	none
17	M	15	10	CPA	7199g>t	G2400V	Central	Trio	de novo	none	none
18	M	12	12	CPA	7423g>t	V2475F	Central	P-M	de novo or P	none	none
19	F	18	8	CPA	11583g>c	Q3861H	Central	Trio	de novo	none	none
20	F	8	8	syncope	11583g>t	Q3861H	Central	Trio	de novo	none	none
21	F	27	6	syncope	11836g>a	G3946S	Central	P-M	de novo or P	none	none
22	F	16	6	syncope	11836g>a	G3946S	Central	Trio	de novo	none	none
23	F	28	28	CPA	11917g>a	D3973N	Central	Trio	Maternal	none	none
24	M	3	3	syncope	12006g>a	M4002I	Central	Trio	de novo	none	none
25	M	11	9	syncope	12371 g>a	S4124N	CT	P-M	Maternal	none	none
26	M	11	11	CPA	12458g>t	S4153I	CT	P-F	de novo or M	none	SD
27	M	11	2	syncope	12533a>g	N4178S	CT	Trio	de novo	none	none
28	F	6	6	CPA	12579c>g	C4193W	CT	Trio	de novo	none	none
29	M	10	10	syncope	13463a>c	Q4488P	CT	Trio	de novo	none	none
30	F	33	9	syncope	13798t>c	F4600L	CT	Trio	de novo	none	none
31	M	28	10	syncope	14174a>g	Y4725C	CT	Trio	de novo	none	none
32	F	23	9	syncope	14311g>a	V4771I	CT	P-M	Maternal	none	syncope
33	M	13	13	CPA	14311g>a	V4771I	CT	P-M	de novo or P	none	none
34	M	17	14	CPA	14806c>a	Q4936K	CT	Trio	de novo	none	none
35	M	5	5	CPA	14834_14835insTCA	4944_4945insH	CT	Trio	de novo	none	none
36	M	12	12	CPA	9910c>g, 14222c>t	Q3304E, A4741V	Central and CT	Trio	Maternal	none	syncope

CPA; cardiac pulmonary arrest, NT; N-terminal, CT; C-terminal, SD; sudden death

**Table 2:** Clinical characteristics of probands with de novo or maternal mutations.

	*de novo*	Maternal
	n = 17	n = 12
Male n (%)	9 (52.9)	4 (33.3)
Mean age of Onset	8.1±3.3	11.0±6.4
CPA n (%)	9 (52.9)	4 (33.3)
Syncope n (%)	8 (47.1)	8 (66.7)
